# Does CRP predict outcome in bipolar disorder in regular outpatient care?

**DOI:** 10.1186/s40345-016-0055-3

**Published:** 2016-07-18

**Authors:** Sonya M. Balukova, Bartholomeus C. M. Haarman, Rixt F. Riemersma-van der Lek, Robert A. Schoevers

**Affiliations:** Department of Psychiatry, CC44, University of Groningen, University Medical Centre Groningen, P.O. Box 30.001, 9700 RB Groningen, The Netherlands

**Keywords:** C-reactive protein, Bipolar disorder, Inflammation, Biological markers, Prognosis, Historic cohort

## Abstract

**Background:**

The association between inflammation and the course of mood disorders is receiving increased attention. This study aims to investigate whether a sub-group of patients with BD can be identified for which a higher CRP (C-reactive protein) level at baseline is associated with an unfavorable prognosis.

**Methods:**

This is a historic cohort study using CRP at baseline, with 15-month follow-up of mood status and medication. Cross-sectional analyses include boxplots, one-way ANOVA, receiver operating characteristics (ROC) curve and Chi square test, and the longitudinal analysis using multivariate Cox-regression.

**Results:**

Eighty-four bipolar disorder patients were included in the analyses. Cross-sectionally, no statistically significant difference was found in CRP distribution across mood states (*p* = 0.372) or rapid cycling state (*p* = 0.656). Also, no CRP cut-off level was distinguished between euthymic and non-euthymic patients according to the ROC curve (*p* = 0.449, AUC = 0.452, 95 % CI 0.327, 0.576), and a literature-derived cut-off value (3 mg/L) again demonstrated no difference (*p* = 0.530). Longitudinally, no association was found between CRP and prognosis of disease neither in euthymic [−2 log likelihood = 120.460; CRP: *p* = 0.866, *B* = −0.011, OR = 0.989 (95 % CI 0.874–1.120)] nor non-euthymic patients [(−2 log likelihood = 275.028; CRP: *p* = 0.802, *B* = 0.010, OR = 1.010 (95 % CI 0.937–1.088)]. Medication use did not affect these associations.

**Conclusions:**

We found no statistically significant association between CRP and a more unfavorable BD prognosis, suggesting that the application of CRP as a practical biomarker to predict outcome in a naturalistic outpatient care setting is not as straightforward as it may seem.

**Electronic supplementary material:**

The online version of this article (doi:10.1186/s40345-016-0055-3) contains supplementary material, which is available to authorized users.

## Background

Bipolar disorder (BD) is associated with a significant decrease in quality of life and social functioning of patients (ten Have et al. 2002). Despite the availability of pharmacological treatment, its efficacy is far from optimal (Burcusa and Iacono 2007; Frecska et al. 2012). A promising approach for optimizing the treatment is to tailor it to the specific characteristics of a patient as part of a ‘personalized medicine’ approach which encompasses not only psychological but also biological markers (Hamdani et al. 2013).

There is increasing evidence to suggest that immunological processes may contribute to the emergence as well as the prognosis and severity of BD (Liu et al. 2004). Apart from pro-inflammatory cytokines such as IL-2 and IL-6 (Brietzke et al. 2009), C-reactive protein (CRP) is an acute phase protein that is produced in response to infection and inflammation. It is also considered to be another candidate biomarker for detecting immune dysregulation in BD. A number of studies suggest an association between CRP and mood disorders, especially during a manic episode (Maes et al. 1995; Dickerson et al. 2007; Cunha et al. 2008).

Based on the idea that immunological processes play a role in the pathophysiology of BD, it can be hypothesized that an increased activity of these processes, measured with CRP, would lead to more instability in BD symptomatology and course of disease. Increased inflammatory activity has been shown to be related to therapy resistance and chronicity in unipolar depression (Sluzewska et al. 1997; Miller et al. 2009; Raison et al. 2013). Recently, Becking et al. (2013) demonstrated an increased CRP to predict future development of manic symptoms in a sample of MDD (major depressive disorder) patients, also suggesting that this is a subtype with an untoward prognosis. However, to date, no studies have examined this issue in bipolar patients.

The current study investigated whether, in a clinical setting, higher CRP levels at baseline may predict a worse BD outcome, defined as a shorter time to relapse (if euthymic) or a longer time to recover. As some medication may influence inflammatory processes, this was taken into account.

## Methods

### Participants and ethical considerations

For the present historic cohort study, we used medical files from 84 BD patients from the BD outpatient department of the Psychiatry Department of the University Medical Center Groningen (UMCG), The Netherlands.

Patients, who provided a written informed consent to participate, were included if the following criteria were fulfilled: DSM-IV-TR diagnosis of BD, age between 18 and 65, recorded CRP value, not pregnant or less than 6 months postpartum, no current serious somatic illnesses (current infections or liver disease, serious un- or undertreated heart, lung or neurological disorders).

All patient data were collected as part of regular outpatient care and were anonymously used for research according to the Data Protection Act (WBP) and Medical Treatment Agreement (WGBO), as formulated in the Code of Conduct for the Use of Data in Health Research, also known as the Research Code of Conduct (see also IRB in Additional file 1).

### Assessments of study parameters

The study parameters included CRP, measurement date and value, BD type, presence of a rapid cycling course, and mood episodes and medication 1 month before and 15 months following the CRP measurement.

Assessment of the psychiatric condition of patients was determined by the first author based on information from two sources: the electronic medical records of the treating psychiatrists and the lifechart methodology (LCM) records (a systematic collection of data on the course of illness and treatment presented in a graphical form) (Denicoff et al. 2000). The condition was noted as one of five categories: (0) Euthymia; (1) Depressive episode; (2) Hypomania or mania; (3) Mixed episode; (4) Unstable mood. Long-lasting clinically significant mood instability that did not fulfill the criteria for any mood episode was assessed as an “unstable” mood, while subthreshold symptoms were assessed as no change in episode (Perlis et al. 2006).

Serum CRP (high sensitive CRP) is routinely measured at hospital admission in this Psychiatry Department (and additional measurements are performed when there is a psychiatric or somatic event for a patient). CRP was assayed using a wide range turbidimetric CRP assay (CRPL3 assay) on a Roche Modular platform (Roche, Mannheim, Germany). Starting from the CRP measurement date at baseline (*T*0), the time to episode change (*T*1) and the corresponding psychiatric condition were gathered from the medical file.

Cases with unclear information and/or diagnosis of current mental status were discussed with the psychiatrists of the BD outpatient department. Remaining decisions were taken in discussion with all authors of this study.

### Statistical analysis

Both cross-sectional and longitudinal analyses were performed. Cross-sectionally, data were visualized using boxplots and tested using histograms, P–P plots, Kolmogorov–Smirnov test, Kruskal–Wallis, a receiver operating characteristics (ROC) curve and Fisher’s exact test.

The longitudinal analysis comprised a multivariate Cox-regression based on the time passed until a change of episode has occurred where the main covariate was the scale of CRP values. This analysis was done separately for euthymic patients and those who were in a mood episode at baseline (depressed, manic, mixed, unstable). This was done because the variable signifying elapsed time has a different meaning for patients in a euthymic state than for a non-euthymic one, and should thus be interpreted differently to describe the progression of disease. Longer time before episode change signifies a better BD prognosis if a subject is euthymic (means longer time in remission), but it means a worse course if a subject is non-euthymic (means longer time in an episode).

Medication was added to the Cox-regression to examine whether it affected CRP or changed the effect of CRP on BD progress. Separate analyses were done after excluding those non-psychopharmaceutical drugs that are known to have anti-inflammatory and/or CRP-affecting properties (O’Brien et al. 2006; Goldstein et al. 2009; Ximenes et al. 2013).

As part of sensitivity analyses, all tests were repeated in each of the following subgroups for each timepoint and then for the whole study: excluding subjects taking anti-inflammatory medication; excluding all medication that affects CRP; excluding outliers, defined as CRP values above 10 mg/L.

## Results

The sample of this study consisted of 84 patients and Table 1 shows their characteristics. Testing the data for normality showed a non-normal distribution of the CRP data, with a positive skewness and a significant difference from a normal distribution seen by Kolmogorov–Smirnov test [D(84) = 0.258, *p* < 0.001]. Because of this, median values are provided for each mood state in Table 1.

**Table 1:** Characteristics of patient population

Parameter	Euthymic	Depressed	(Hypo)manic	Mixed	Unstable	All
Mean age (SD)	44.9 (12.4)	42.7 (12.4)	43.2 (10.3)	46 (7.1)	40.5 (10.2)	43.6 (11.7)
Number of subjects (%)	37 (44.0)	27 (32.1)	12 (14.3)	2 (2.4)	6 (7.1)	84 (100)
Male gender (%)	11 (37.9)	10 (34.5)	4 (13.8)	2 (6.9)	2 (6.9)	29 (34.5)
Female gender (%)	26 (47.3)	17 (30.9)	8 (14.5)	0	4 (7.3)	55 (65.5)
Number of subjects with BD type I (%)^a^	26 (41.9)	21 (33.9)	11 (17.7)	2 (3.2)	2 (3.2)	62 (100)
Number of subjects with BD type II (%)^a^	7 (41.2)	5 (29.4)	1 (5.9)	0	4 (23.5)	17 (100)
Number of subjects with rapid cycling (%)	4 (30.8)	3 (23.1)	2 (15.4)	0	4 (30.8)	13 (100)
Number of subjects with metabolic syndrome (%)^b^	9 (13.8)	6 (9.2)	2 (3.1)	1 (1.5)	2 (3.1)	20 (30.8)^c^
Median C-reactive protein (mg/L)	1.10	2.10	0.90	3.08	1.39	1.37

*SD* standard deviation, *BD* bipolar disorder

^a^Bipolar disorder type information missing for five patients

^b^Metabolic syndrome information missing for 19 patients (criteria: fasting glucose ≥5.6 mmol/L and two or more of following: BMI ≥30 kg/m^2^, hypertriglyceridemia ≥1.7 mmol/L, HDL-C <0.9 mmol/L in men and <1.0 mmol/L in women, hypertension ≥140/90 mmHg)

^c^The number in brackets in this cell represents the percentage of all subjects who have metabolic syndrome, while the rest of the percentages in this row represent the distribution of subjects with metabolic syndrome across the mood states

### Cross-sectional analysis

**Fig. 1:**
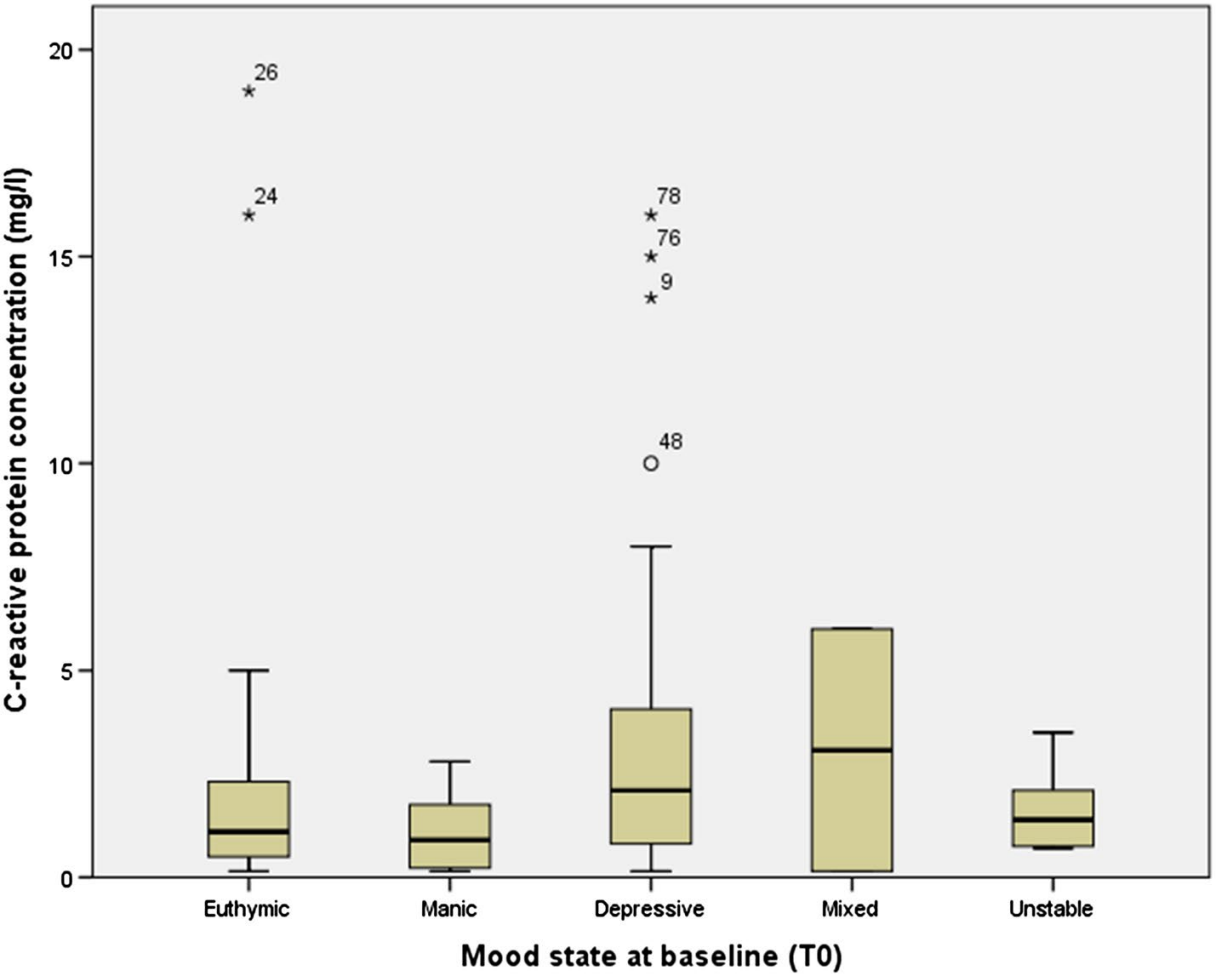
Boxplot of CRP distribution at baseline across mood state. The *Y*-axis depicts CRP concentration in mg/L starting from 0 mg/L and incrementing with 5 mg/L. In the *X*-axis, *bars* signify the interquartile range of CRP values in each mood group, and the *thickened line* inside them shows the median value of CRP within this mood group. The *stars* and *circle* show the boxplot outliers, while the *numbers* next to them signify the specific number of the patient with this outlier value. The *circle* is a patient with CRP value closest to the threshold value but above it, so it is also not included

**Fig. 2:**
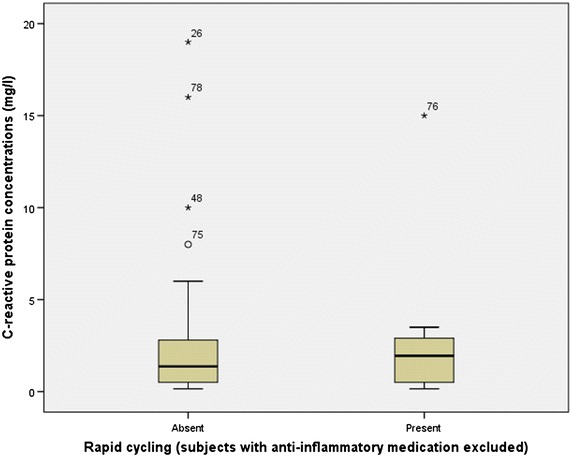
Boxplot of CRP distribution between patients with and without rapid cycling. The *Y*-axis depicts CRP concentration in mg/L starting from 0 mg/L and incrementing with 5 mg/L. In the *X*-axis, *bars* signify the interquartile range of CRP values in each patient group, and the *thickened line* inside them shows the median value of CRP within this mood group. The *stars* and *circle* show the boxplot outliers, while the *numbers* next to them signify the specific number of the patient with this outlier value

The differences between the distribution of CRP values among the mood states were first examined using Kruskal–Wallis. No statistical significance was found (*p* = 0.372) (see Table 1). Figure 1 shows the distribution of mood episodes at baseline. Using Kolmogorov–Smirnov, no statistical significant difference was found between the patients that were rapid cycling and patients that were not (*p* = 0.656, see Fig. 2). Repeating the cross-sectional analyses excluding all medications that are known to affect CRP, as well as excluding CRP outliers, generally yielded the same, non-significant results.

After excluding six CRP outliers, there were 37 (44 %) who were in euthymia and 47 (56 %) were non-euthymic: 27 (32.1 %) were in a depressive episode, 12 (14.3 %) in a manic episode, 2 (2.4 %) in a mixed episode and 6 (7.1 %) were unstable.

Using an ROC curve, the data were tested to identify a cut-off value of CRP which could suggest whether subjects at baseline would be euthymic or in an episode. Based on the curve (Additional file 1: Figure S1), no such cut-off value was found (AUC = 0.452, *p* = 0.449, 95 % CI 0.327, 0.576).

Consequently, the cross-sectional analyses were performed using a literature-based CRP cut-off value of 3.0 mg/L and a Chi square test for the not-normal distribution of CRP value. The results showed that, cross-sectionally, higher CRPs were almost equally distributed among euthymic and non-euthymic patients (42.1 % euthymic and 57.9 % non-euthymic by CRP > 3 mg/L; 44.6 % euthymic and 55.4 % non-euthymic by CRP ≤ 3 mg/L). Fisher’s exact test confirmed that these results have no statistical significance (*p* = 0.530).

### Longitudinal analysis

#### For euthymic subjects at baseline

**Fig. 3:**
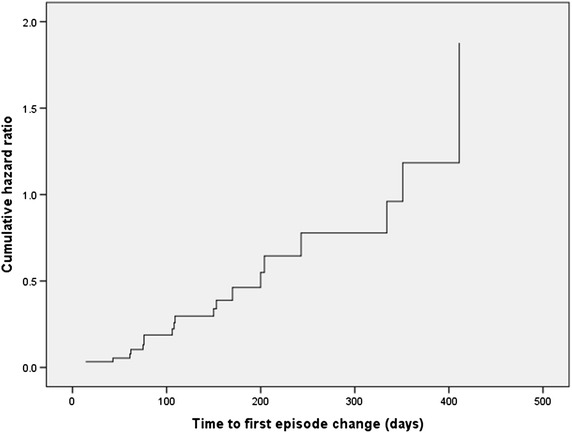
Hazard function at mean of the covariate CRP for subjects euthymic at baseline. This figure illustrates what the hazard ratio is for relapsing of a subject with a given CRP value compared to a subject with a CRP value of 1 unit lower in the course of the studied period. The *Y*-axis represents the rate of relapsing to a non-euthymic state of all subjects euthymic at baseline. Elapsed time period in days until a change in the mood state has occurred (to either manic, depressed, mixed state or unstable) is depicted on the *X*-axis

From the 37 subjects who were euthymic at baseline, 20 had a change of episode during the trial period, while 17 patients stayed euthymic. The results are shown in Table 2 and the Hazard function of the covariate CRP is in Fig. 3. There is no correlation between CRP value and the event of episode change (relapsing). The odds ratio (=0.989) approaches equality for both groups (results are very close to the neutral line). Moreover, these findings are not statistically significant and so the null hypothesis could not be rejected.

**Table 2:** Longitudinal results from Cox-regression

Patient group	CRP after adjusting for					
	−2 log likelihood	Sig.	B coef.	Odds ratio	95 % CI of odds ratio	
					Lower	Upper
Euthymic patients at baseline	120.460	0.866	−0.011	0.989	0.874	1.120
Non-euthymic patients at baseline	275.028	0.802	0.010	1.010	0.937	1.088

#### For non-euthymic subjects at baseline

**Fig. 4:**
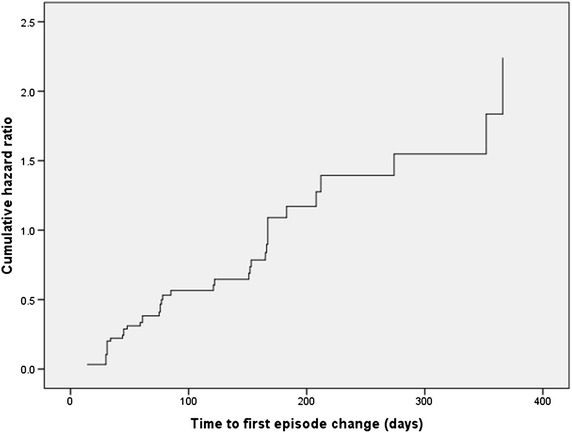
Hazard function at mean of the covariate CRP for subjects sick at baseline. This figure illustrates what the hazard ratio is for recovering of a subject with a given CRP value compared to a subject with a CRP value of 1 unit lower in the course of the studied period. The *Y-axis* represents the rate of recovering of all subjects sick at baseline. The elapsed time period in days until an euthymic state has occurred is depicted in the *X-axis*

There were 47 subjects who were in an episode at baseline of which 30 became euthymic, while 17 patients remained in a mood episode during the study period. The results are shown in Table 2 and in Fig. 4. The odds ratio is approaching equality (results are very close to the neutral line) and these findings are not statistically significant. As seen in the euthymic group, these results also do not show an association between CRP and relapsing.

After adjusting for covariates and testing for interactions of CRP with medication at the two timepoints, there were no results with statistical significance in each of the two mood-state groups—euthymic and non-euthymic. Adjusting for separate medication types used in any moment during the study again yielded no associations with statistical significance.

Table 3 shows a summary of the used medication at the *T*0 and *T*1 timepoints as well as in total for the whole study period.

**Table 3:** Number of subjects per medication type

Parameter	*T*0^a^	*T*1^b^	Total^c^
Lithium (%)	31 (36.9)	47 (56)	48 (57.1)
Valproic acid (%)	14 (16.7)	9 (10.7)	16 (19.0)
Anti-inflammatory drugs (%)^d^	12 (14.3)	12 (14.3)	16 (19.0)
Tricyclic antidepressants (TCA) (%)^d^	5 (6)	5 (6)	7 (8.3)
Antidepressants (non-TCA) (%)	31 (36.9)	27 (32.1)	32 (38.1)
Antipsychotics (%)	35 (41.7)	34 (40.5)	39 (46.4)
Benzodiazepines (%)	31 (36.9)	31 (36.9)	39 (46.4)

^a^Medication used up to and including the baseline (*T*0)

^b^Medication used up to and including the time of episode change is such occurred (*T*1)

^c^Medication used for the total period of the study (1 month before and up to 15 months after CRP measurement, unless episode change occurred earlier)

^d^Values are the same for both *T*0 and *T*1 timepoints

## Discussion

### Principal findings

To our knowledge, this is the first study examining longitudinal associations between CRP level and clinical outcome in BD patients in a naturalistic treated and real-life measurement outpatient setting. In a first cross-sectional analysis, we could not distinguish a sub-group of BD patients with an elevated baseline CRP level based on affective state or rapid cycling state. In the longitudinal analysis, no statistically significant association was found between higher CRP values and relapsing in either euthymic or non-euthymic patients, as well as when comparing them.

### Comparison to previous studies

The results of the cross-sectional analysis can be compared to several previous studies on the association of CRP with BD. Two of the studies with a total of 202 patients reported that for their BD subjects higher CRP was significantly associated with manic state compared to the other mood episodes (Dickerson et al. 2007; Cunha et al. 2008).

Three other studies with a total of 248 patients, however, did not demonstrate significant differences in CRP across the mood states, which is corresponding to the current study results (Hornig et al. 1998; Hope et al. 2011; Tsai et al. 2012).

The findings of the influence of medication on the reported associations between CRP values and mood states in the above studies are also conflicting. Hornig et al. (1998) found a significant negative interaction between lithium and CRP within BD patients, while Dickerson et al. (2007) found that no medication affected the CRP association with BD, as we found in the current study.

In a recent meta-analysis Dargél et al. (2015) demonstrated an overall cross-sectional association between BD and CRP. In this meta-analysis, CRP levels were elevated in manic and euthymic patients compared to HC, but not in depressed BD patients compared to HC. Recent prospective longitudinal studies demonstrated CRP alterations across mood states (Jacoby et al. 2016), as well as before and after various treatments (Uyanik et al. 2015). In addition, an increased CRP was found to be associated with an increased risk for developing late-onset BD in a large Danish demographic sample (Wium-Andersen et al. 2016). Adding to this body of knowledge, the present study shows that using CRP as a practical biomarker to predict outcome in a naturalistic outpatient care setting is not as easily applicable as it may seem.

## Limitations

The present study has several limitations. A limitation pertaining the use of historical data is that CRP measurements were more or less routinely measured, and more in those patients who were psychiatrically or somatically ill. Since it was not purely measured as a routine, a selection bias cannot be ruled out.

Furthermore, body fat percentage, smoking, blood pressure, physical activity and SNP polymorphisms are suggested to be associated with different baseline levels of CRP (Palosuo et al. 1986; Visser et al. 1999; Ford 2002; Davey Smith et al. 2005; Marnell et al. 2005) and in the present study, we were not able to correct for these variations.

Finally, the study would have benefitted from a larger sample size.

### Proposed mechanisms

There are two factors to consider which could play a role in shaping the results from this study: use of medication and the pathophysiological model of BD.

Due to the naturalistic design of this study, a great variety of medications was used, some of which have been suggested to have anti-inflammatory properties. Lithium, part of the standard treatment of dipolar disorder in the Netherlands, was used by more than half of the patients in this study (57.1 %). Although its mechanism of action in bipolar depression is not fully understood yet (Goldstein et al. 2009), several studies have found evidence that lithium causes a decrease in the inflammatory marker CRP (Sluzewska et al. 1997; Hornig et al. 1998) and a similar effect is also described for SSRIs (selective serotonin reuptake inhibitors) used by 38 % of the subjects here (18). However, there are reports that lithium and antidepressants may also exhibit pro-inflammatory properties (Goldstein et al. 2009; Hamer et al. 2011; Nassar and Azab 2014). These properties were postulated after observing the stimulating effect of lithium on pro-inflammatory cytokines such as Tumor Necrosis Factor-α (TNF-α), Interleukin-4 (IL-4) and Interleukin-6 (IL-6). It is not yet clear what causes these reverse effects. There could be various mechanisms via which medication is influencing CRP. However, at the same time no such effect was demonstrated in this study. Therefore, other factors should be considered.

Our findings therefore suggest that the inflammatory model of BD is probably much more complex than what can be shown by straightforward inflammatory alterations demonstrated by elevated markers such as CRP (Altamura et al. 2014) at one point in time.

Compared to the inflammatory mechanism in somatic disorders where CRP, being an acute phase protein, directly correlates to acute worsening of a disease, it may be that immunological processes in BD are affected by more factors and additional mechanisms, which need to be added to the model to gain a better understanding. One such hypothesis is that inflammatory changes in psychiatric disorders are mediated through a shift toward the tryptophan/kynurenine pathway, which leads to the formation of further factors that may affect the functioning of the brain, such as neurotoxic accumulation of the metabolite 3-hydroxykynurenine (Myint and Kim 2013). Another hypothesis is the co-occurrence of autoimmunity alongside immune dysregulation in BD. It is thus likely that BD is related to a state of immune dysregulation, rather than more pure immune activation (Rege and Hodgkinson 2013; Haarman et al. 2014).

It has been suggested that the immune-mediating pathogenesis of BD occurs at a much younger age, and what follows is a dysregulation of the bodily systems. The long-lasting results are then what we see at an older age: unhealthy and unexpected interactions and responses of the whole body, and of inflammatory markers as well (Beumer et al. 2012). Another interesting possibility is that what we see is not necessarily an inflamed but a damaged organism, given that CRP reacts not only to inflammatory stimuli, but also to damaged cells (Kushner et al. 2006).

To increase our understanding of the position of immune-system-mediated pathophysiological processes in BD, it is necessary to measure CRP and other relevant immune bio-assays prospectively in a larger sample size. In that regard, it is important to investigate what happens on an intra-individual level: additional trials are necessary measuring CRP on different timepoints in a prospective longitudinal manner so that individual changes could be followed and analyzed. Perhaps, these studies will elucidate models, adjusting for variables known to influence CRP, that eventually enable CRP to be used as a practical biomarker to predict outcome in naturalistic treatment settings.

## Additional file

10.1186/s40345-016-0055-3 Supplemental information containing additional IRB approval information and **Figure S1**: ROC curve of CRP tested for euthymic vs. non-euthymic subjects.

## Abbreviations

BD: bipolar disorder
CRP: C-reactive protein
LCM: lifechart methodology
MDD: major depressive disorder
