# Supplementary material for: Does CRP predict outcome in bipolar disorder in regular outpatient care?
Source: Int J Bipolar Disord. 2016 Jul 18;4:14. doi: 10.1186/s40345-016-0055-3 (PMC4949199; doi:10.1186/s40345-016-0055-3)

**Supporting information**

IRB Approval

In accordance with the Data Protection Act (the Dutch WBP) and Medical Treatment Agreement (the Dutch WGBO), as formulated in the Code of Conduct for the Use of Data in Health Research (known as the Research Code of Conduct), IRB approval was not necessary and not sought as all patient data were collected as part of regular outpatient care and were anonymously used for research. All patients provided written informed consent to anonymous use of their data for these analyses.

Supplemental figure S1

***Figure S1 ROC curve of CRP tested for euthymic vs. non-euthymic subjects***


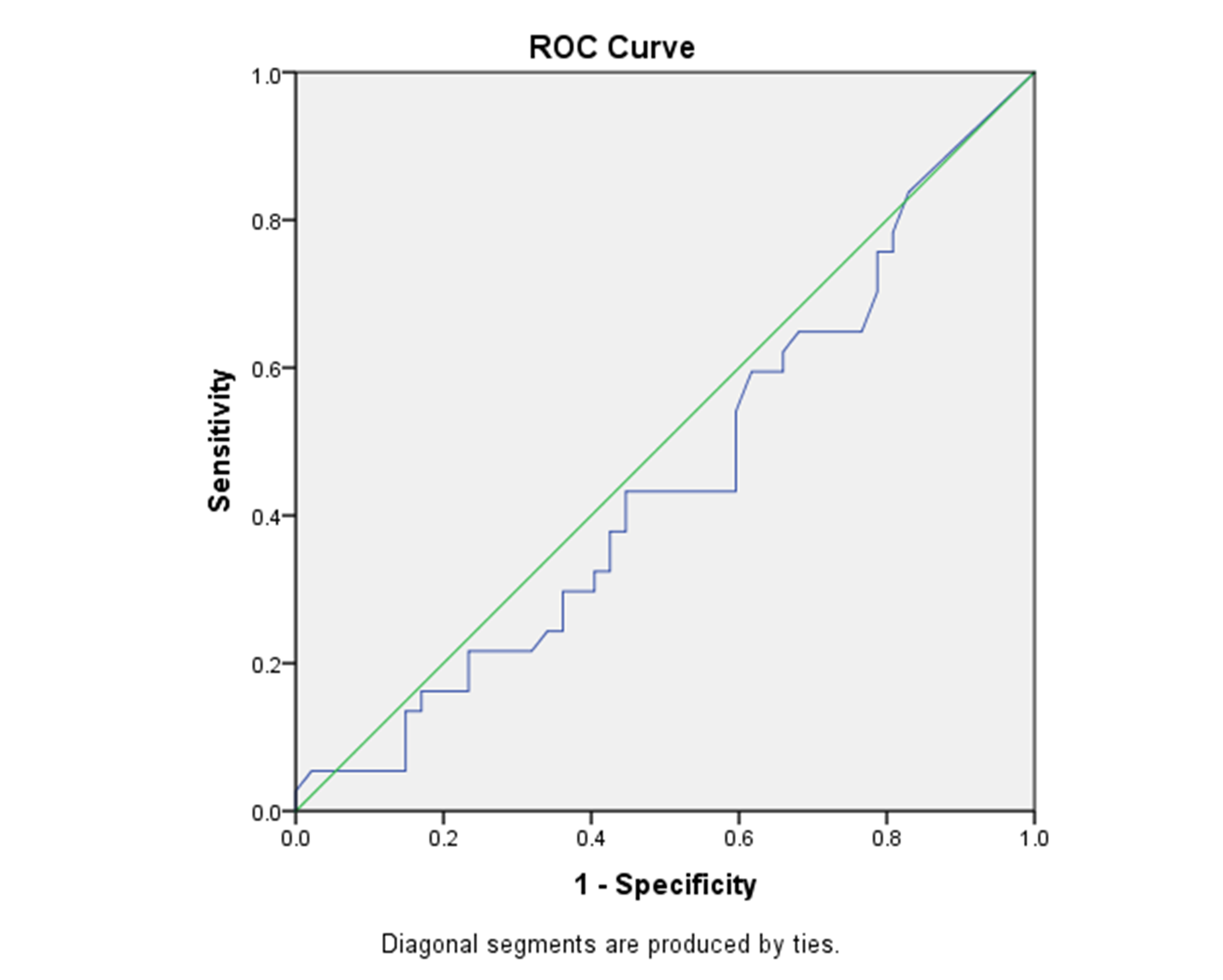

Supplement: Supplementary file 1 — 10.1186/s40345-016-0055-3 Supplemental information containing additional IRB approval information and Figure S1: ROC curve of CRP tested for euthymic vs. non-euthymic subjects. [file 40345_2016_55_MOESM1_ESM.doc]
